# Comparative Analysis of Time-Series Forecasting Models for eLoran Systems: Exploring the Effectiveness of Dynamic Weighting

**DOI:** 10.3390/s25144462

**Published:** 2025-07-17

**Authors:** Jianchen Di, Miao Wu, Jun Fu, Wenkui Li, Xianzhou Jin, Jinyu Liu

**Affiliations:** School of Electrical Engineering, Naval University of Engineering, Wuhan 430033, China; 21100602@nue.edu.cn (J.D.); fjsd@21cn.com (J.F.); lwk_nue@163.com (W.L.); jxz2065629079@163.com (X.J.); jinyuliu0531@163.com (J.L.)

**Keywords:** eLoran system, ASF prediction, deep learning, system performance optimisation, LSTM, random forest

## Abstract

**Highlights:**

**What are the main findings?**

- The dynamic weighting model achieves the highest prediction accuracy among all methods.
- It effectively balances accuracy and computational efficiency for time-series forecasting.
- The model enables accurate real-time ASF prediction in enhanced Loran systems.
- It offers a practical solution for forecasting in resource-constrained navigation systems.

**What is the implication of the main finding?**

- The high prediction accuracy of the dynamic weighting model indicates its potential to improve the performance of time-series forecasting tasks in various fields, especially in navigation systems where accuracy is crucial.
- The balance between accuracy and computational efficiency makes it suitable for application in resource-constrained environments, providing a feasible approach to address the challenges of forecasting in such settings.
- Enabling accurate real-time ASF prediction in enhanced Loran systems can enhance the reliability and effectiveness of these navigation systems.
- Offering a practical solution for forecasting in resource-constrained navigation systems contributes to the development and application of navigation technologies in limited resource scenarios.

**Abstract:**

This paper presents an advanced time-series forecasting methodology that integrates multiple machine learning models to improve data prediction in enhanced long-range navigation (eLoran) systems. The analysis evaluates five forecasting approaches: multivariate linear regression, long short-term memory (LSTM) networks, random forest (RF), a fusion model combining LSTM and RF, and a dynamic weighting (DW) model. The results demonstrate that the DW model achieves the highest prediction accuracy while maintaining strong computational efficiency, making it particularly suitable for real-time applications with stringent performance requirements. Although the LSTM model effectively captures temporal dependencies, it demands considerable computational resources. The hybrid model utilises the strengths of LSTM and RF to enhance the accuracy but involves extended training times. By contrast, the DW model dynamically adjusts the relative contributions of LSTM and RF on the basis of the data characteristics, thereby enhancing the accuracy while significantly reducing the computational demands. Demonstrating exceptional performance on the ASF2 dataset, the DW model provides a well-balanced solution that combines precision with operational efficiency. This research offers valuable insights into optimising additional secondary phase factor (ASF) prediction in eLoran systems and highlights the broader applicability of real-time forecasting models.

## 1. Introduction

### 1.1. Background of the Enhanced Long-Range Navigation System

Enhanced long-range navigation (eLoran) is a terrestrial radio navigation system that represents an advanced version of the conventional Loran-C system. Unlike satellite-based navigation systems such as GPS and GLONASS, eLoran provides positioning and navigation services to maritime, terrestrial, and airborne users using low-frequency signals transmitted from terrestrial stations [1,2,3,4,5]. Operating typically at approximately 100 kHz, eLoran signals offer strong penetration capabilities and wide coverage, enabling reliable navigation in environments where global navigation satellite system (GNSS) signals are weakened or obstructed by dense physical barriers, severe weather, or intentional interference [6,7].

eLoran is particularly advantageous for positioning and navigation in areas with limited GNSS coverage or where GNSS performance is compromised. Its robust anti-jamming capabilities make it a reliable alternative to GNSS, especially critical for the protection of essential infrastructure [8,9,10]. However, eLoran faces specific limitations—most notably, its lower positioning accuracy compared to GNSS. This issue is especially prominent in maritime environments, where multipath effects, ionospheric disturbances, and atmospheric conditions adversely affect accuracy. Accordingly, enhancing the positioning precision through the accurate prediction and correction of additional secondary phase factor (ASF) data has become a central focus in both academic and industrial research [11].

### 1.2. Overview of the eLoran System and ASF Prediction

As a modern enhancement of the Loran-C system, eLoran determines positional coordinates by measuring the time difference between radio signals transmitted from terrestrial stations and received by user equipment [12]. In contrast to satellite-based systems, eLoran exhibits superior anti-jamming resilience and reliability, particularly in challenging settings such as urban canyons, indoor facilities, and underground environments. These characteristics make it a compelling solution for ensuring continuous and dependable navigation in scenarios where satellite signals are unavailable [13].

The ASF is a pivotal parameter that directly affects the positioning accuracy of the eLoran system [14]. It reflects the system’s adaptive response to environmental changes. In practice, ASF values influence signal quality and stability, being affected by meteorological conditions, topographical variations, and the technical limitations of the equipment. The accurate forecasting of ASF variations allows for the early identification of potential signal degradation, enabling the eLoran system to dynamically adjust its parameters in real time to sustain optimal navigation performance and reliability [15,16,17,18,19].

However, predicting the ASF values remains a complex task because of the numerous interacting factors involved, including the terrain elevation, surface characteristics, climatic conditions, and signal propagation paths. These variables contribute to the inherent difficulty and unpredictability in ASF forecasting.

Extensive research has been devoted to developing methods for ASF correction and prediction. For example, Wang et al. developed an ASF correction method using backpropagation neural networks (BPNN) combined with transfer learning [12]. Initially trained on theoretical ASF data, the BPNN model was subsequently fine-tuned through transfer learning to accommodate real-world uncertainties. This approach reduced the average absolute prediction error from 2.02 μs to 0.29 μs, thereby substantially enhancing the positioning accuracy [20].

Park et al. further validated the effectiveness of BPNN-based ASF correction, emphasising the necessity of rigorous calibration across diverse operational environments [3]. Their findings were supported by real-world data and highlight the potential of advanced machine learning algorithms to significantly improve the accuracy of Loran-based navigation systems.

Additionally, Kim et al. highlighted the vulnerability of GNSS to frequency interference [2], prompting the establishment of Korea’s eLoran testbed system in 2021. They also explored novel ASF surveying methods in narrow waterways to refine the ASF map precision. Park et al. investigated multichain Loran positioning techniques and demonstrated that excluding outliers from ASF corrections significantly improved the positioning accuracy [18]. Son et al. evaluated the performance of the eLoran system, addressing its potential and limitations, and emphasised the critical role of ASF research in enhancing navigation reliability. They also pointed out the specific challenges in accurately surveying ASF in narrow channels, identifying this as an ongoing research issue [11].

Conventional methods for predicting ASF primarily rely on empirical and physical models, which often fail to address the nonlinear complexities inherent in ASF data [21]. With the increasing availability of large datasets and advancements in computational resources, machine learning-based predictive models have gained traction. However, individual machine learning models frequently face limitations in terms of prediction accuracy and generalisability. Consequently, the integration of multiple models has emerged as a preferred strategy for addressing the multifaceted challenges associated with ASF prediction.

In summary, accurate ASF prediction is critical to improving the positioning accuracy of eLoran systems. Existing methods often struggle to effectively capture and adapt to dynamic ASF variations [22]. Recent research has increasingly emphasised data-driven models that proactively improve the positioning accuracy by forecasting changes in ASF values. Although a variety of time-series prediction models are extensively applied in navigation data forecasting, each model has distinct strengths and limitations that necessitate careful selection based on specific environmental conditions. In this paper, we propose a comprehensive approach that integrates multiple machine learning models, including long short-term memory (LSTM) networks, random forest (RF), a fusion model, and a dynamic weighting (DW) model. Through a comparative analysis, we identify the most effective predictive strategy customised to the unique data characteristics of the eLoran system.

## 2. Model Construction

The proposed ASF prediction framework consists of three main components: data acquisition and preprocessing, the construction of a deep learning model, and the development and integration of a fusion-based learning approach. In the data acquisition and preprocessing phase, we collected multisource data, including the station signal field strength, signal-to-noise ratio, packet perimeter difference, arrival times, and historical ASF values. We then performed preprocessing tasks such as data cleaning, normalisation, and feature engineering to ensure a high-quality dataset suitable for model training [23].

For the deep learning model, we adopted LSTM as the foundational architecture. As a specialised form of recurrent neural network (RNN), LSTM is well suited for capturing long-term dependencies in time-series data, making it particularly effective for the temporally correlated nature of ASF prediction [24]. We designed a multilayer LSTM network comprising input layers, multiple LSTM hidden layers, and fully connected output layers. By fine-tuning key parameters—including the number of layers, neuron counts, and activation functions—we optimised the model’s predictive performance.

For the construction and integration of the learning model, we selected RF as the base model. RF is an ensemble learning technique that enhances generalisation and model stability by constructing multiple decision trees and aggregating their predictions [25]. We developed an RF model composed of numerous decision trees and optimised its performance by fine-tuning key parameters such as the number of trees and their maximum depth.

To further improve the prediction accuracy, we introduce two distinct model fusion strategies that integrate the predictive outputs of the LSTM and RF models. The specific fusion approaches are described in detail in the subsequent subsections.

### 2.1. Multiple Linear Regression Model (Physical)

Multiple linear regression (MLR) was employed in this study as a classical statistical forecasting method [26]. The core principle of MLR is to model the trend of the dependent variable by linearly combining multiple independent variables (features). In this study, MLR was used as a benchmark model—referred to as “physical”—to evaluate the validity of an explicit linear relationship between the features and the target variable. The mathematical formulation of MLR is

(1) $$y=\beta_{0}+\beta_{1}x_{1}+\beta_{2}x_{2}+\cdots +\beta_{n}x_{n}+\epsilon ,$$

where *y* is the target variable (ASF1 or ASF2), $x_{1},x_{2},\ldots ,x_{n}$ represent the input features (where n∈{6,12,20}), $\beta_{0}$ is the intercept term, and $\beta_{1},\beta_{2},\ldots ,\beta_{n}$ denote the regression coefficients. ϵ is the residual term, assumed to follow a normal distribution with a mean of 0. Here, *m* denotes the number of observations in the dataset.

The parameters were estimated using ordinary least squares (OLS), with the objective of minimising the sum of squares of the residuals:

(2) $$\underset{\beta }{\min}\sum^{\begin{array}{c} i=1\\ m \end{array}}{\left(y_{i}-\sum^{\begin{array}{c} j=0\\ n \end{array}}\beta_{j}x_{ij}\right)}^{2}.$$

MLR is a foundational method in regression analysis that establishes a linear relationship between the input and target variables. A separate regression model was trained for each target variable.

The performance metrics included the mean square error (MSE), mean absolute error (MAE), and correlation coefficient (R).

### 2.2. Long Short-Term Memory Network

#### 2.2.1. Overview of the LSTM Model

LSTM networks are a specialised type of RNN designed to address the gradient vanishing problem commonly encountered in conventional RNNs. This aim is achieved through a gating mechanism that allows LSTM networks to effectively capture and retain long-term dependencies in time-series data [27]. The fundamental architecture of LSTM comprises three types of gates—forget gates, input gates, and output gates—that regulate the flow of information through the network.

#### 2.2.2. Mathematical Formalisation

Let the input of time step *t* be $x_{t}\in {\mathbb{R}}^{d}$, the hidden state be $h_{t}\in {\mathbb{R}}^{h}$, and the cell state be $C_{t}\in {\mathbb{R}}^{h}$; then, the LSTM is computed as follows.

① Oblivion gate—controls the proportion of historical information retained:

(3) $$f_{t}=\sigma \left(W_{f}\cdot \left[h_{t-1},x_{t}\right]+b_{f}\right)$$

② Input gate—determines the amount of new information stored:

(4) $$i_{t}=\sigma \left(W_{i}\cdot \left[h_{t-1},x_{t}\right]+b_{i}\right)$$

(5) $${\tilde{C}}_{t}=\tanh\left(W_{C}\cdot \left[h_{t-1},x_{t}\right]+b_{C}\right)$$

③ Cell state renewal:

(6) $$C_{t}=f_{t}⊙C_{t-1}+i_{t}⊙{\tilde{C}}_{t}$$

④ Output gate—generates the current hidden state:

(7) $$o_{t}=\sigma \left(W_{o}\cdot \left[h_{t-1},x_{t}\right]+b_{o}\right)$$

(8) $$h_{t}=o_{t}⊙\tanh\left(C_{t}\right)$$

where σ is the sigmoid function with an output range of [0, 1], ⊙ denotes element-wise multiplication, $W_{f},W_{i},W_{C},W_{o}$ are the trainable weight matrices, and $b_{f},b_{i},b_{C},b_{o}$ denote the bias terms.

#### 2.2.3. Key Characteristics

Gating mechanism: This utilises sigmoid-generated signals for precise information control.

Gradient stability: The linear transfer of cell states helps to solve the issue of vanishing gradients, ensuring more stable learning dynamics.

### 2.3. Random Forest

#### 2.3.1. Overview of the RF Model

RF is a decision tree-based ensemble learning algorithm that employs the bagging technique to improve model generalisation and reduce the risk of overfitting. This approach involves constructing multiple decision trees during training and aggregating their predictions, typically through majority voting for classification or averaging for regression tasks [28]. The RF algorithm introduces two key sources of randomness to enhance model robustness: feature randomness, which involves selecting random subsets of features at each split, and sample randomness, which is achieved through bootstrap sampling from the training dataset.

#### 2.3.2. Mathematical Principles

① Decision tree splitting criterion

At each node m of the decision tree, a feature *j* and a split threshold *s* are selected to maximise the information gain. The criteria for splitting vary by task. Here, *K* is the number of classes, and *k* indexes each class. $N_{m}$ denotes the total number of samples at each node; $N_{\mathrm{left}}$ and $N_{\mathrm{right}}$ are the numbers of samples in the left and right child nodes, respectively.

(a) Gini coefficient (categorisation task):

(9) $$Gini\left(D_{m}\right)=1-\sum_{k=1}^{K}{\left(\frac{\left|C_{k}\right|}{\left|D_{m}\right|}\right)}^{2}$$

Gini gain after splitting:

(10) $$\Delta Gini=Gini\left(D_{m}\right)-\frac{N_{\mathrm{left}}}{N_{m}}Gini\left(D_{\mathrm{left}}\right)-\frac{N_{\mathrm{right}}}{N_{m}}Gini\left(D_{\mathrm{right}}\right)$$

(b) Mean square error (regression task):

(11) $$MSE\left(D_{m}\right)=\frac{1}{N_{m}}\sum_{i\in D_{m}}{\left(y_{i}-{\bar{y}}_{m}\right)}^{2}$$

where ${\bar{y}}_{m}$ is the sample mean at node *m*.

② Random forest construction

(a) Input: A training set $D={\left\{\left(x_{i},y_{i}\right)\right\}}_{i=1}^{N}$, number of trees *T*, and the size of the feature subset *m_try_*.

(b) Process: For *t* = 1 to *T*, Generate a bootstrap sample *D_t_* from *D* by sampling with replacement; Train the decision tree *f_t_* by selecting the optimal split from *m_try_* randomly chosen features at each split.

(c) Output: For regression tasks, the output is the mean prediction from all trees; for classification tasks, it is the majority vote.

(12) $$\hat{y}=\frac{1}{T}\sum_{t=1}^{T}f_{t}(x)$$

### 2.4. Fusion Modelling Approach

The fusion modelling approach includes LSTM feature extraction, LSTM feature outputs, RF regression, and final fusion.

#### 2.4.1. LSTM Feature Extraction

The LSTM network processes the input time-series data to extract features, which are high-level representations used to capture long-term dependencies [29]. The formula for feature extraction at time step *t* is

(13) $$h_{t}=LSTM\left(x_{t},h_{t-1}\right),$$

where $x_{t}$ is the input feature at time *t*, $h_{t}$ is the hidden state at time step *t*, and $h_{t-1}$ is the hidden state from the previous time step.

#### 2.4.2. LSTM Feature Outputs

In LSTM networks, the hidden state from the last time step (or alternatively from selected parts of the sequence) is typically used to represent features of the entire input sequence. In the proposed method, the features extracted using the LSTM layer are used as input in the RF model.

#### 2.4.3. Random Forest Regression

Subsequent to feature extraction by the LSTM, these features are fed into an RF regression model. The RF model predicts the target variable by aggregating the outputs of multiple decision trees.

The predictive formula is

(14) $${\hat{y}}_{RF}=\frac{1}{T}\sum_{i=1}^{T}{\hat{y}}_{i},$$

where *T* is the number of trees in the forest and ${\hat{y}}_{i}$ represents the prediction by the *i*-th tree. The final prediction of the RF represents the average of the predictions across all trees.

#### 2.4.4. Final Fusion Prediction Results

The output of the fusion model is a weighted average of the regression predictions derived from the features extracted by the LSTM and RF:

(15) $${\hat{y}}_{\mathrm{fusion}}=w_{\mathrm{lstm}}\cdot {\hat{y}}_{lstm}+w_{rf}\cdot {\hat{y}}_{rf},$$

where ${\hat{y}}_{\mathrm{lstm}}$ is the prediction by the LSTM network, ${\hat{y}}_{\mathrm{rf}}$ is the prediction from the RF model, and $w_{\mathrm{lstm}}$, $w_{\mathrm{rf}}$ denote the weight coefficients for the LSTM and RF models, respectively.

These weights are typically determined through training or cross-validation, ensuring that their sum equals 1 to balance the contributions of both models effectively.

### 2.5. LSTM-RF Fusion Method Based on Dynamic Weight Optimisation

#### 2.5.1. Overview of the Method

To address the challenge of dynamic changes in data distribution inherent in time-series prediction tasks, the proposed method introduces a fusion model that combines LSTM and RF with dynamically optimised weights. This approach enhances the prediction accuracy and stability in complex environments by enabling the real-time adjustment of the fusion weights between the two model types. By integrating the temporal modelling strengths of LSTM with the robust feature handling capabilities of RF, the model delivers more reliable and accurate forecasts.

#### 2.5.2. Dynamic Weight Optimisation Mechanism

##### Sliding-Window Dynamic Optimisation

Algorithmic processes:

① Inputs: Historical data $D_{\mathrm{train}}$, validation window length $T_{w}$, base model (LSTM with RF)

② Initialisation:

Training base models: $M_{lstm}\leftarrow LSTM\left(D_{\mathrm{train}}\right),M_{rf}\leftarrow \mathrm{RandomForest}\left(D_{\mathrm{train}}\right)$

Initial weights $\left(w_{1}^{\left(0\right)},w_{2}^{\left(0\right)}\right)$, determined by grid search on the validation set.

③ Sliding Update:

For each time window t=1,2,…,

(a) Get new data window $D_{val}^{\left(t\right)}$;

(b) Calculate prediction error:

(16) $$E_{l\;\mathrm{stm}}^{\left(t\right)}=\frac{1}{T_{w}}\sum_{i=1}^{T_{w}}\left|y_{i}-{\hat{y}}_{\mathrm{lstm}}^{\left(i\right)}\right|$$

(17) $$E_{rf}^{\left(t\right)}=\frac{1}{T_{w}}\sum_{i=1}^{T_{w}}\left|y_{i}-{\hat{y}}_{rf}^{\left(i\right)}\right|$$

(c) Update weights:

(18) $$E_{rf}^{\left(t\right)}=\frac{1}{T_{w}}\sum_{i=1}^{T_{w}}\left|y_{i}-{\hat{y}}_{rf}^{\left(i\right)}\right|$$

④ Output: Sequence of dynamic weights $\left\{\left(w_{1}^{\left(t\right)},w_{2}^{\left(t\right)}\right)\right\}$

Features: This method balances computational efficiency and model fitness through periodic local reoptimisation.

##### Online Gradient Descent Optimisation

① Mathematical formalisation:

Define dynamic weight optimisation as an online learning problem with constraints.

Objective function—minimise cumulative prediction loss:

(19) $$\begin{array}{c} \underset{w_{1},w_{2}}{\min}\sum_{k=1}^{T}{\left(y_{k}-\left(w_{1}{\hat{y}}_{lstm}^{\left(k\right)}+w_{2}{\hat{y}}_{rf}^{\left(k\right)}\right)\right)}^{2}\\ \;s.t.w_{1}+w_{2}=1,\;w_{1}\geq 0,w_{2}\geq 0 \end{array}$$

② Iterative update steps:

(a) Initialise the weights: $w_{1}^{\left(0\right)}=0.5,w_{2}^{\left(0\right)}=0.5$;

(b) For each time step k=1, … ,K, perform the following.

Note: The variable *k* here refers to the iteration index in the online optimisation process and is independent of the class index *k* used in Equation (9).

I Receiver-based model predictions:

(20) $${\hat{y}}_{lstm}^{\left(k\right)},{\hat{y}}_{rf}^{\left(k\right)}$$

II Calculate fusion prediction:

(21) $${\hat{y}}_{\mathrm{fused}}^{\left(k\right)}=w_{1}^{\left(k\right)}{\hat{y}}_{lstm}^{\left(k\right)}+w_{2}^{\left(k\right)}{\hat{y}}_{rf}^{\left(k\right)}$$

III Calculate gradient:

(22) $$\begin{array}{c} \frac{\partial L^{\left(k\right)}}{\partial w_{1}}=-2\left(y_{k}-{\hat{y}}_{fused}^{\left(k\right)}\right){\hat{y}}_{l\;\mathrm{stm}}^{\left(k\right)}\\ \frac{\partial L^{\left(k\right)}}{\partial w_{2}}=-2\left(y_{k}-{\hat{y}}_{\mathrm{fused}}^{\left(k\right)}\right){\hat{y}}_{rf}^{\left(k\right)} \end{array}$$

IV Update weights:

(23) $$\begin{array}{c} w_{1}^{\left(k+1\right)}=w_{1}^{\left(k\right)}-\eta \frac{\partial L^{\left(k\right)}}{\partial w_{1}}\\ w_{2}^{\left(k+1\right)}=w_{2}^{\left(k\right)}-\eta \frac{\partial L^{\left(k\right)}}{\partial w_{2}} \end{array}$$

V Project onto feasible domain:

(24) $$w_{1}^{\left(k+1\right)},w_{2}^{\left(k+1\right)}\leftarrow Proj_{S}\left(w_{1}^{\left(k+1\right)},w_{2}^{\left(k+1\right)}\right)$$

including

(25) $$S=\left\{\left(w_{1},w_{2}\right)|w_{1}+w_{2}=1,w_{i}\geq 0\right\}$$

③ Parameter setting: The learning rate *η* is determined by cross-validation, with an initial recommended value of 0.01.

##### Weight Update Strategy in the Dynamic Weighting Model

The DW model updates the weights of its base learners (LSTM and RF) according to their real-time performance. Specifically, the prediction errors (e.g., absolute error or MAE) at each time step are used to compute dynamic weights using a softmax-like exponential function:

(26) $$w_{i}(t+1)=\frac{\exp\left(-\alpha \cdot e_{i}(t)\right)}{\sum_{j}\exp\left(-\alpha \cdot e_{j}(t)\right)},$$

where $e_{i}(t)$ is the error of model *i* at time *t*, and α is a sensitivity factor controlling the adjustment intensity.

To prevent instability caused by short-term fluctuations or noise, we apply exponentially weighted moving average (EWMA) smoothing to the weights and constrain them within a defined range (e.g., [0.2, 0.8]) to maintain ensemble diversity.

#### 2.5.3. Integration of Forecasting Frameworks

The overall implementation process of this method is as follows.

① Base model training phase:

Independently train LSTM and RF models using historical data.

Generate predictive sequences on the validation set ${\left\{{\hat{y}}_{lstm}^{\left(i\right)}\right\}}_{i=1}^{N}\;$ and $\;{\left\{{\hat{y}}_{rf}^{\left(i\right)}\right\}}_{i=1}^{N}$.

② Dynamic weight optimisation phase:

Select dynamic strategies based on the application scenarios (sliding window/online learning).

Calculate weight sequences according to the algorithm described in Section Sliding-Window Dynamic Optimisation or Section Online Gradient Descent Optimisation.

③ Real-time forecasting phase:

For moment *t*, obtain the base model prediction: ${\hat{y}}_{lstm}^{\left(t\right)},{\hat{y}}_{rf}^{\left(t\right)}$.

Calculate the dynamic weights: $w_{1}^{\left(t\right)},w_{2}^{\left(t\right)}$.

Output fusion predictions: ${\hat{y}}_{\mathrm{fused}}^{\left(t\right)}=w_{1}^{\left(t\right)}{\hat{y}}_{l\mathrm{stm}}^{\left(t\right)}+w_{2}^{\left(t\right)}{\hat{y}}_{rf}^{\left(t\right)}$.

Specifically, the DW model dynamically adjusts the weights on the basis of the prediction errors (such as MAE) of the LSTM and RF models in the sliding window or at the current time. The lower the prediction error of a model in a sliding window or the current timestep, the higher the weight it receives. This approach is implemented using two optimisation strategies: ① a sliding window-based weight update mechanism and ② an online gradient descent approach with projection into a feasible domain. Additionally, we introduce the use of EWMA and range constraints (weights between 0.2–0.8) to prevent instability and abrupt changes. This approach allows the DW model to adaptively emphasise the model that is more effective under the current data characteristics while maintaining prediction stability.

## 3. Materials and Methods

### 3.1. Data Preprocessing

**Figure 1:**
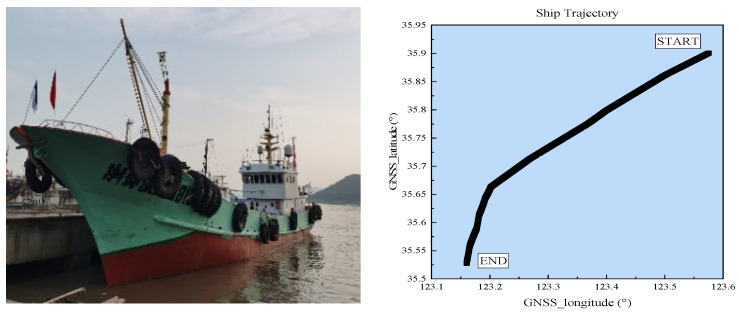
Test vessel and navigation route map.

The data utilised in this study were obtained from actual measurements of the eLoran system at sea. The specific dataset corresponds to sailing activities in the Qingdao Sea area, China, recorded between 07:00 and 10:00 on 30 September 2024, under clear weather conditions. The sailing route and test vessel are depicted in Figure 1.

The dataset for this analysis consisted of 10,048 records and encompassed the following features:

- Signal strength;
- Signal-to-noise ratio;
- Positioning accuracy;
- GNSS positioning data;
- Timestamps indicating the exact moments of data collection.

The primary variables for prediction, ASF1 and ASF2, were identified as the targets. The preprocessing steps undertaken to prepare the dataset included the following.

① Data cleansing

Checking missing values: Linear interpolation was employed to fill gaps in the data. Segments containing more than ten consecutive missing values were removed.

Rejection of outliers: According to the 3σ principle, data points that fell beyond three standard deviations from the mean were discarded. Extreme outliers, such as zero signal strength or negative signal-to-noise ratios, were directly excluded.

② Data alignment

The fields of station strength, signal-to-noise ratio, packet perimeter difference, signal arrival time, ASF1, and ASF2 were synchronised using timestamps to ensure temporal consistency. For data with misaligned timestamps, nearest-neighbour interpolation was applied for proper alignment.

③ Normalisation

**Figure 2:**
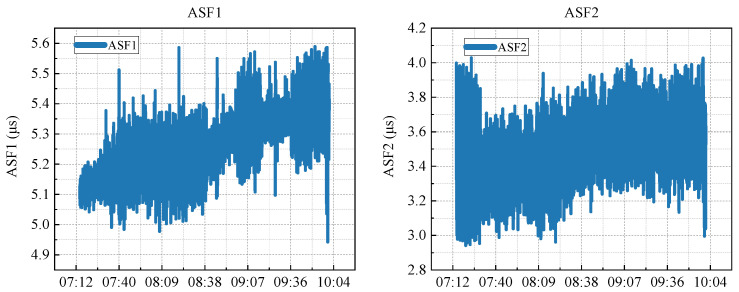
Visualisation of the actual ASF1 and ASF2 sequences in the dataset.

To reduce the impact of feature magnitude disparities on the model’s weight distribution, *Z*-score normalisation was conducted on all features. This normalisation process transforms the data to a uniform scale with a mean of 0 and a standard deviation of 1:

(27) $$z=\frac{x-\mu }{\sigma },$$

where *x* is the original data value, *μ* is the mean of the dataset, *σ* is the standard deviation, and *z* is the normalised value. Post-normalisation, all feature values ranged between 0 and 1. A comparative analysis of ASF1 and ASF2 as shown in Figure 2, before and after normalisation, was conducted as follows.

Although the forecasting task in this study was regression-based rather than classification-based, the distribution of the target variables (ASF1 and ASF2) still played a critical role in model performance. To assess the presence of distributional imbalance or skewness in the dataset, we performed the following analyses.

**Figure 3:**
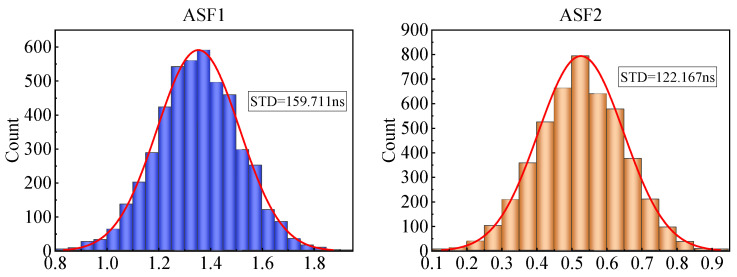
Histograms of the propagation delay distribution.

We plotted histograms of the ASF1 and ASF2 values, as shown in Figure 3. The distributions showed slight positive skewness, with most values clustered around the mean but with a small tail toward higher values.

To address potential influences from extreme values, we applied a 3σ-based outlier removal strategy, which helped to reduce the distribution tails while preserving the main structure of the data.

Although the degree of skewness was acceptable, we acknowledge that nonuniform target distributions can still introduce bias during training. Therefore, we applied Z-score normalisation to standardise the scales of both the inputs and targets and ensured that the training–validation–test splits covered diverse operational conditions.

In future work, we plan to explore weighted loss functions or quantile-based sampling strategies to further mitigate distribution bias and enhance the prediction robustness.

**Figure 4:**
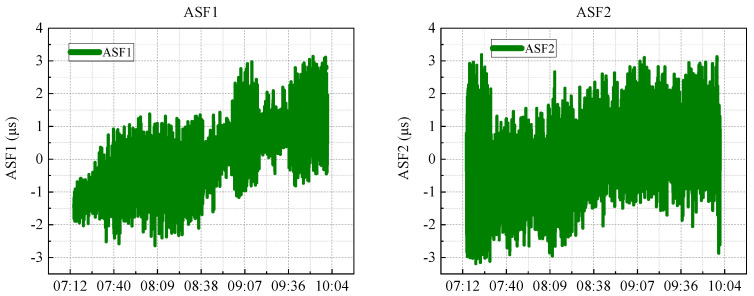
Visualisation of the normalised ASF1 and ASF2 sequences in the dataset.

Figure 4 illustrates Z-score normalisation rather than bounding to a fixed range. Using descriptive analysis, it was observed that the ASF values typically fluctuated within a specific range, with the majority of the data points clustering around a defined interval. During the data cleansing phase, initial attention was directed toward addressing missing values. A modest number of missing entries was identified in the ASF1 and ASF2 columns (149 and 164 records, respectively) and they were imputed using linear interpolation. Outliers, detected using the Z-score method, were adjusted when they exceeded three standard deviations from the mean by replacing them with values closer to the norm.

To reduce the effects of differing feature scales, Z-score normalisation was applied to all variables. This task involved subtracting the mean and dividing by the standard deviation for each feature, thereby standardising them to a common scale. This normalisation step ensures that the model training process remains unbiased by disparities in feature magnitude.

### 3.2. Model Design and Implementation

The dataset used in this study included 20 features and two target variables. Preprocessing involved separating features from targets and splitting the data chronologically into training (70%), validation (15%), and test (15%) sets. The features and targets were then formatted to meet the input requirements of each model, with specific training and evaluation protocols implemented accordingly.

In this study, five predictive models were evaluated: MLR (referred to as “physical”), LSTM, RF, a fusion model, and a DW model. The detailed design parameters and training configurations for each model are described in this section.

#### 3.2.1. Physical Multiple Linear Regression Model

The MLR model establishes a linear relationship between the input and target variables. Model training was conducted using the fitlm function in MATLAB (R2023b), with the performance evaluated using metrics such as the MSE, MAE, and correlation coefficient (R). Although MLR models are computationally efficient and quick to train, they typically perform poorly on complex time-series data because of their inherent linearity.

#### 3.2.2. LSTM Networks

LSTM is a variant of the RNN that excels in modelling and predicting sequence data, making it well suited for time-series analysis. In this study, LSTM was employed to forecast the target variables from time-series data.

① Model structure
  (a) Input layer: Accepts time-series data with 20 features.
  (b) LSTM layer: Comprises 128 cells that process timing information.
  (c) Dropout layer: To prevent overfitting, set to 0.3.
  (d) Fully connected layer: Outputs a single predicted value.
  (e) Regression layer: Used for regression tasks, outputs continuous values.
② Training configuration
  (a) Optimiser: Adam optimiser.
  (b) Maximum training cycle: 100 rounds.
  (c) Validation data: Validation using validation sets.
  (d) Batch size: Adaptive.

**Figure 5:**
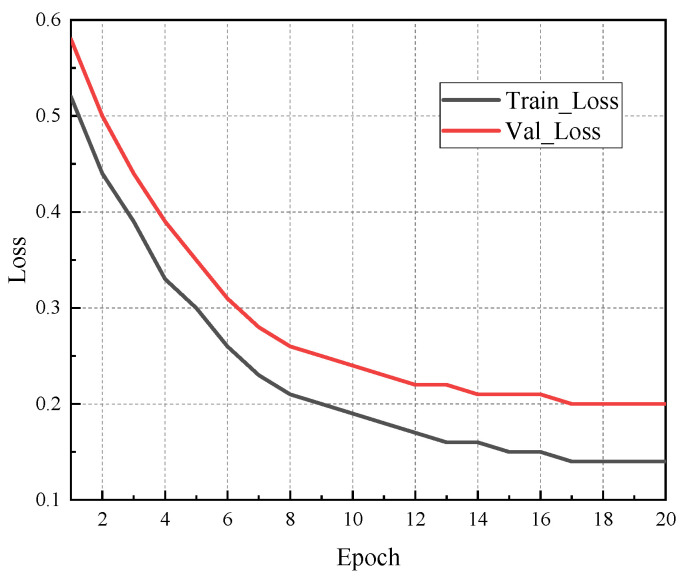
Training and validation loss curve of the LSTM model (ASF2).

To mitigate the risk of overfitting in the LSTM model, we employed a combination of early stopping and dropout mechanisms during training. A dropout rate of 0.3 was applied to the LSTM layer, and early stopping was triggered if the validation loss did not improve over 10 consecutive epochs. Moreover, five-fold cross-validation was conducted on the training dataset. The results demonstrated consistent performance across folds, indicating the model’s robustness. Figure 5 presents a comparison of the training and validation loss over epochs, showing convergence and a minimal performance gap. These measures effectively improved the model’s generalisation capability while preventing overfitting.

#### 3.2.3. Random Forest

RF is an integrated learning method that makes predictions using multiple regression trees. We used an RF model with 200 trees, which enabled out-of-bag (OOB) data prediction to avoid overfitting. The model performed better when dealing with nonlinear relationships.

#### 3.2.4. Fusion Model

The fusion model integrates the deep learning capabilities of LSTM with the ensemble benefits of RF, utilising both for enhanced predictive accuracy.

① Model structure

LSTM layer: Identical to the standalone model described previously.

Random forest: Trains on features derived from the LSTM to conduct regression predictions.

② Training process
  (a) Train the LSTM model to extract features.
  (b) Train an RF regression model on the features extracted by LSTM for prediction.

**Figure 6:**
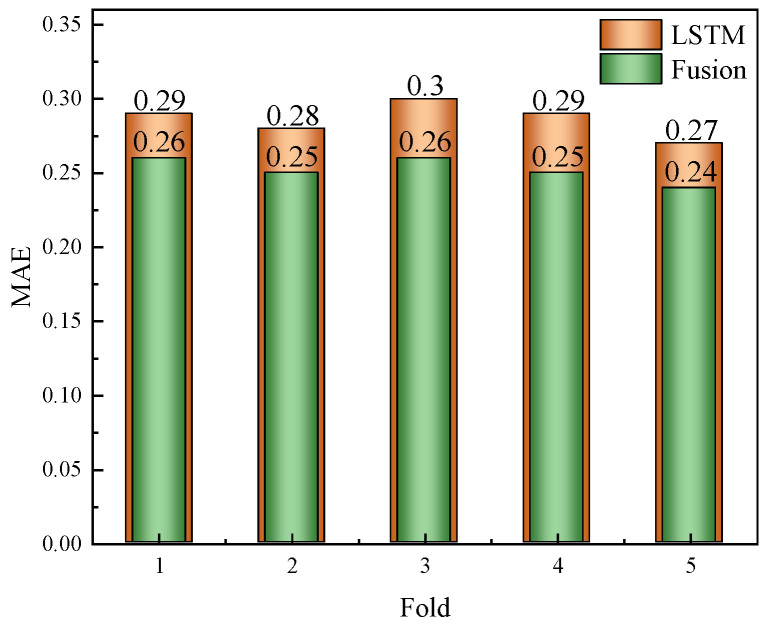
Validation MAE comparison across five folds for the LSTM and fusion models.

The fusion model, integrating LSTM feature extraction with RF regression, also underwent overfitting assessment using five-fold cross-validation. We monitored the MSE and MAE on both the training and validation sets across folds. Similarly to the LSTM model, early stopping was employed for the LSTM component, whereas the RF component leveraged out-of-bag (OOB) error estimates. As shown in Table 1 and Figure 6, the variance in the validation performance across folds was low, and the training–validation error gap remained within an acceptable range. This result indicates that the fusion model generalises well to unseen data, without significant overfitting.

Table 1 summarises the results of the five-fold cross-validation for the LSTM and fusion models on the ASF2 dataset. The differences between the training and validation metrics were minimal, and the validation R scores remained consistently high, indicating that both models generalised well. The fusion model slightly outperformed the LSTM in most cases.

Although the fusion model demonstrated strong predictive accuracy, its training time posed limitations for time-sensitive applications. To address this issue, we implemented several optimisations and evaluated their effects.

① Simplified LSTM architecture: We reduced the number of hidden units from 128 to 64 and limited the LSTM to a single layer. This modification shortened the training time by approximately 35%, while maintaining predictive accuracy with a minor increase in the MAE (approximately 2.8%) and negligible impact on the R value.

② Dimensionality reduction before RF: A principal component analysis (PCA)-based feature reduction step was introduced between the LSTM and RF components. This modification reduced the input dimensionality for the RF model, resulting in an approximately 20% shorter training time for the RF stage, without affecting the accuracy.

③ Parallel training pipeline: We modified the model training routine to allow for the concurrent training of the LSTM and RF modules when feasible, significantly decreasing the overall wall-clock training time.

Table 2 shows a comparison of the original and optimised fusion models in terms of the training time and validation performance. These results suggest that the optimised fusion architecture provides a promising tradeoff between predictive accuracy and efficiency.

**Figure 7:**
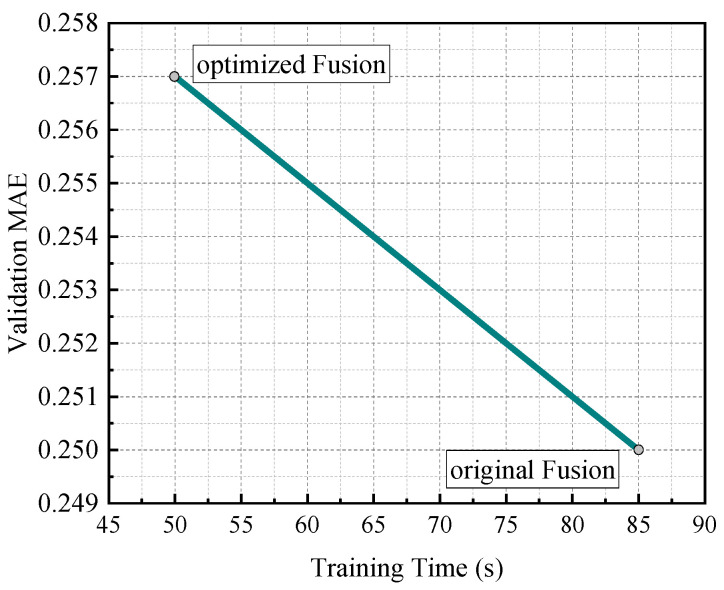
Tradeoff between training time and prediction accuracy for original and optimised fusion models.

Figure 7 illustrates the relationship between the training time and validation mean absolute error (MAE).

① The original fusion model achieved higher predictive accuracy (MAE ≈ 0.250) but required a longer training time (approximately 85 s).

② The optimised fusion model significantly reduced the training time (approximately 50 s), with only a slight decrease in accuracy (MAE ≈ 0.257).

These results indicate that, by simplifying the model architecture and introducing parallel optimisation, the training efficiency can be substantially improved while maintaining high predictive performance.

#### 3.2.5. Dynamic Weighting Model

DW models combine the advantages of LSTM and RF models by dynamically adjusting the weights for fusion prediction. The main idea is to determine the contribution of each model to the prediction on the basis of the models’ performance at each moment in time (using an error metric such as the MAE). Specifically, to improve the accuracy of the final prediction, the model dynamically tunes the weights of each model according to the variations in error, particularly the MAE, of each model.

① Error calculation: In the proposed DW model, at each time step, the MAEs of the LSTM and RF models are calculated. The MAE reflects the average difference between the predicted and true values. Typically, models with smaller errors are assigned higher weights.

② Weight update: The weight of each model is calculated dynamically by comparing the MAEs of the LSTM and RF models:

(28) $$w_{\mathrm{new}}=\frac{1/MAE_{LSTM}}{\left(\frac{1}{MAE_{LSTM}}+\frac{1}{MAE_{RF}}\right)},$$

where $w_{\mathrm{new}}$ is the new weight, and $MAE_{LSTM}$ and $MAE_{RF}$ denote the mean absolute errors of the LSTM and RF models, respectively.

③ EWMA: To reduce abrupt fluctuations in model weights, EWMA smoothing is employed. This method smooths the weight adjustments over time by blending the current and previous weights, controlled by a smoothing factor α. The weight update formula is

(29) $$w_{t}=\alpha \cdot w+(1-\alpha )\cdot w_{\mathrm{previous}},$$

where $w_{t}$ denotes the weight of the current time step; α is the smoothing factor, whose value is between 0 and 1; and $w_{\mathrm{previous}}$ is the weight of the previous moment.

④ Weight constraints: To avoid excessive weight changes or a model occupying excessive weight, constraints are used to limit the range of weight values. In the proposed model, the minimum value of the weights was set to 0.2 and the maximum value to 0.8—that is,

(30) $$0.2\leq w_{t}\leq 0.8.$$

Final prediction: From the calculated weights, the final fusion prediction is generated by combining the predictions of the LSTM and RF:

(31) $$y_{\hat{t}}=w_{t}\cdot y_{LSTM}+\left(1-w_{t}\right)\cdot y_{RF},$$

where $y_{\hat{t}}$ is the final predicted value, and $y_{LSTM}$ and $y_{RF}$ denote the predicted values for LSTM and RF, respectively.

⑤ Smoothing factor (α): The degree to which the current weights are influenced by the previous weights is regulated by α. A higher α value, closer to 1, results in more significant weight adjustments based on the most recent data, whereas a lower α, closer to 0, smooths the weight changes more substantially. In this study, α was set between 0.1 and 0.3, adjusted on the basis of the experimental results to balance model stability with responsiveness to error fluctuations.

To assess the impact of the smoothing factor α on the DW model’s performance, we conducted experiments with varying values of α = {0.05, 0.10, 0.20, 0.30, 0.50} on both the ASF1 and ASF2 datasets. The results are summarised in Table 3.

When α was too small (e.g., 0.05), the weight updates were too slow to adapt to real-time error variations, leading to suboptimal prediction accuracy. Conversely, a large α (e.g., 0.50) caused weight oscillations and instability in the predictions. The best performance was achieved at α = 0.20, which yielded the lowest MAE and highest R on both datasets (ASF1: MAE = 0.079, R = 0.976; ASF2: MAE = 0.084, R = 0.970).

This analysis confirmed that the DW model was robust to the choice of α within a reasonable range and that careful tuning can significantly enhance the performance.

⑥ Window size: The window size is calibrated on the basis of the time-series characteristics. Larger window sizes help to smooth out fluctuations, whereas smaller windows more sensitively reflect rapid changes in the data.

⑦ Weight constraints: To prevent instability in the final predictions, the weights were constrained to a predefined range. This approach ensures that neither model is overly or insufficiently weighted, which can skew the results. In this study, the weights were set to maintain a minimum of 0.2 and a maximum of 0.8, promoting a balanced contribution from both the LSTM and RF models.

The DW model continuously adjusts the weights of each submodel in real time, using the strengths of both the LSTM and RF models. This method is particularly advantageous in scenarios where one model may outperform the other at different times. By dynamically optimising the model fusion based on the actual performance, this approach offers flexibility and adaptability.

**Figure 8:**
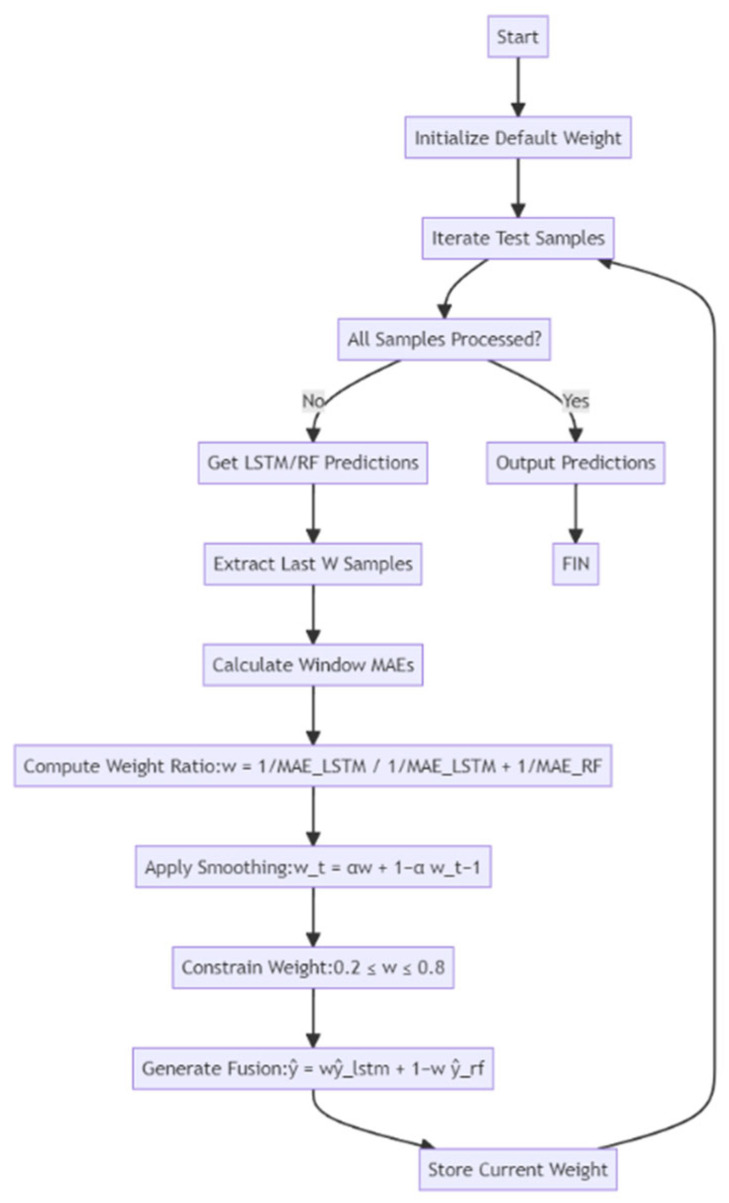
Block diagram of the dynamic weighting fusion model design and parameter configuration.

Additionally, the model strikes a balance between computational efficiency and prediction accuracy by using an appropriate smoothing factor. Unlike static weighting strategies that depend solely on one model, dynamic weighting provides a versatile and adaptable prediction method, well suited to the evolving patterns and discrepancies observed in time-series data. A block diagram showing the DW fusion model design and the parameter configuration is shown in Figure 8.

### 3.3. Hyperparameter Optimisation Strategy

To ensure optimal model performance, we adopted a two-step hyperparameter optimisation strategy. First, a coarse grid search was used to identify suitable ranges for key parameters. Subsequently, fine-tuning was conducted manually on the basis of the five-fold cross-validation performance. 

LSTM: A grid search was performed over the number of hidden units (64, 128, 256), dropout rates (0.2, 0.3, 0.5), learning rates (0.001, 0.005), and batch sizes (16, 32, 64). The best configuration (128 units, 0.3 dropout, 0.001 learning rate) was selected according to the validation MAE.

RF: We optimised the number of trees (100 to 500), maximum depth (10 to 50), and minimum samples per leaf (1, 3, 5) using a randomised search strategy, with five-fold cross-validation on the training set.

Fusion and DW models: Hyperparameters were inherited from the best-performing LSTM and RF configurations. Additionally, the DW model’s smoothing factor (α = 0.1 to 0.3) and window size (10 to 50) were tuned using a grid search.

All search processes used the validation MAE as the evaluation criterion, and the final settings were selected to balance accuracy and computational efficiency.

## 4. Evaluation and Results

In this study, we determined the performance of five distinct predictive models on time-series data from the eLoran system, using a variety of evaluation metrics, such as the MSE, MAE, R, and training time. Additionally, to illustrate the models’ performance more clearly, we present plots of each model’s predictions alongside the actual values and visualisations of the dynamic weight changes in the LSTM model.

The assessment was conducted using the following evaluation metrics.

① Mean squared error (MSE)

Measures the average squared difference between predicted and actual values:

(32) $$MSE=\frac{1}{n}\sum_{i=1}^{n}{\left(y_{i}-{\hat{y}}_{i}\right)}^{2}$$

The MSE is an absolute error metric, expressed as the square of the unit of the target variable (μs^2^).

② Mean absolute error (MAE)

Represents the average of the absolute differences between predicted and actual values:

(33) $$MAE=\frac{1}{n}\sum_{i=1}^{n}\left|y_{i}-{\hat{y}}_{i}\right|$$

The MAE is also an absolute metric, with the same unit as the target variable (μs).

③ Correlation coefficient (R)

Indicates the strength of the linear relationship between the predicted and actual values. It is a relative, unitless metric ranging from −1 to 1, where values closer to 1 indicate stronger correlations.

④ Training Time

The total time required for model training, measured in seconds (s).

### 4.1. Forecasting Accuracy

To ensure consistency and comparability across different datasets and evaluation scenarios, we further computed normalised metrics including the normalised MAE (nMAE) and normalised RMSE (nRMSE). These metrics are defined as

(34) $$nMAE=\frac{MAE}{\sigma_{y}},\;nRMSE=\frac{\sqrt{MSE}}{\sigma_{y}},$$

where $\sigma_{y}$ is the standard deviation of the original target variable (ASF1 or ASF2). These values are presented alongside the original metrics in Table 4 and Table 5.

The physical model demonstrated the weakest performance across all target variables. Notably, for the ASF1 dataset, it exhibited a high MSE of 168,265.57 and a correlation coefficient (R) of only 0.13, highlighting its inadequate fit and inability to capture meaningful patterns in the data. Although its MSE improved to 37.62 on the ASF2 dataset, it still performed substantially worse than the other models, failing to achieve satisfactory predictive accuracy. Although computationally efficient, the physical model falls short of the accuracy standards required for practical applications.

The LSTM model showed notable improvements by effectively capturing long-term dependencies inherent in the time-series data. For the ASF1 dataset, it achieved an MSE of 2.21, an MAE of 0.73, and an R value of 0.44, reflecting moderate predictive performance with some residual errors and variability. The model performed better on the ASF2 dataset, with an MSE of 0.84, an MAE of 0.37, and an R value of 0.61. These results confirm the LSTM model’s capability to model temporal dependencies, although its prediction error remains higher compared to the RF and fusion models.

The RF model demonstrated superior performance, particularly in modelling nonlinear relationships, which are common in regression tasks. On the ASF1 dataset, the RF model achieved an MSE of 0.37, an MAE of 0.46, and an R value of 0.73, indicating robust predictive accuracy. Its performance was further enhanced on the ASF2 dataset, with an MSE of 0.24, an MAE of 0.27, and an R value of 0.87. These outcomes highlight the RF model’s effectiveness in capturing complex data patterns and its strong generalisation capabilities.

The fusion model combines the feature extraction capabilities of the LSTM with the regression strengths of the RF model, resulting in enhanced predictive accuracy. On the ASF1 dataset, the fusion model achieved an MSE of 0.51, an MAE of 0.50, and an R value of 0.69, demonstrating notable improvements over the standalone LSTM and RF models, albeit at the cost of longer training times. On the ASF2 dataset, it was further improved, registering an MSE of 0.21 and an MAE of 0.25 and maintaining an R value of 0.87—delivering the most accurate predictions among all models evaluated. By effectively utilising the complementary strengths of LSTM and RF, the fusion model significantly enhances both the regression performance and prediction accuracy.

The DW model offers an optimal balance between predictive accuracy and computational efficiency by dynamically adjusting the weights assigned to the LSTM and RF models. On the ASF1 dataset, the DW model recorded an MSE of 0.48, an MAE of 0.47, and an R value of 0.68, positioning it among the top-performing models. Although it did not outperform the RF model in terms of the MSE and MAE, it provided superior computational efficiency. For the ASF2 dataset, the DW model was further improved, achieving an MSE of 0.35, an MAE of 0.26, and an R value of 0.79, demonstrating enhanced prediction accuracy and efficient data processing. By dynamically adjusting the weighting of predictions, the DW model integrates the advantages of both LSTM and RF, achieving a well-balanced tradeoff between accuracy and computational efficiency, particularly excelling in scenarios with strict real-time constraints. Model deployment resource assessment is shown in Table 6.

### 4.2. Computational Efficiency

Computational efficiency is a critical consideration, especially when processing large-scale datasets or performing real-time predictions, where the model training time plays a significant role. This section provides a detailed analysis of the computational efficiency of each predictive model.

The primary advantage of the physical model lies in its simplicity, resulting in an exceptionally short training time of just 0.055 s. This high computational efficiency makes it well suited for applications requiring rapid real-time performance, particularly when computational resources are limited. However, its low predictive accuracy makes it unsuitable for tasks demanding high precision.

In contrast, the LSTM model, with a training time of 73.91 s, reflects the substantial computational demands of its deep learning architecture. Training the LSTM involves optimising a large number of parameters to effectively model time-series data, which significantly increases its computational burden, especially in the context of large datasets. Although the LSTM model excels in capturing long-term temporal dependencies, its extended training time may limit its suitability for time-sensitive or resource-constrained prediction tasks.

Conversely, the RF model achieved a training time of 7.28 s, significantly faster than the LSTM model. This efficiency is attributed to its architecture, which constructs multiple decision trees and aggregates their outputs. Although more computationally intensive than simpler models, RF provides an effective balance between computational efficiency and predictive accuracy, making it a practical choice for a wide range of applications.

The fusion model, which combines the LSTM and RF models, exhibited a substantially longer training time of 99.76 s. The simultaneous training of both component models significantly increases the computational demand, with the LSTM component being the primary contributor to this overhead. Although the fusion model offers superior predictive accuracy, its extended training time poses limitations for real-time applications and environments with restricted computational resources.

In contrast, the DW model demonstrated exceptional computational efficiency, completing training in just 0.02 s—substantially faster than all other models. This remarkable performance highlights the DW model’s ability to maintain high prediction accuracy while significantly enhancing its computational efficiency through dynamic weight adjustments. It not only matched the RF and fusion models in predictive accuracy but also exceeded them in efficiency, making it particularly well suited for real-time prediction scenarios.

**Figure 9:**
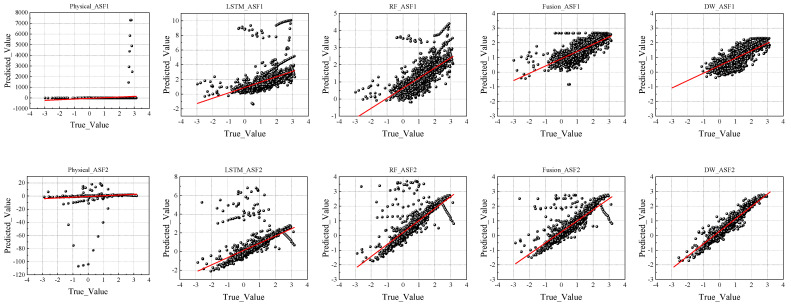
Forecast comparison across different models.

To further illustrate the predictive performance of each model, we compared their predicted values with actual observations, as shown in Figure 9.

In the prediction comparison chart presented in Figure 9, the physical model exhibits a notably weak correlation between the predicted and actual values. The scatterplot shows a widespread distribution of points with no clear pattern, and a significant portion of the predictions deviate markedly from the true values. This dispersion underscores the model’s limited predictive utility and inability to capture underlying data trends.

In contrast, the scatterplots for the LSTM and RF models exhibit stronger alignment between the predicted and actual values, particularly in the ASF2 dataset, where the RF model demonstrated a superior fit. The fusion and DW models further refined the prediction accuracy, with the predicted values clustering more tightly around the diagonal line, indicating a higher degree of correlation. In particular, the DW model’s scatterplot reveals fewer outliers and a more consistent linear trend, highlighting its robustness and superior handling of data irregularities.

**Figure 10:**
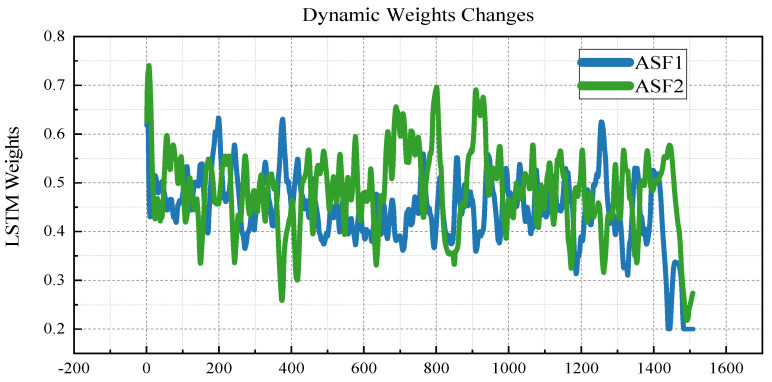
Variations in dynamic weight adjustments over time.

Figure 10 illustrates the dynamic adjustment of the LSTM model weights over time during the prediction process. The observed fluctuations in the LSTM weight values across timesteps emphasise the importance of the dynamic weighting mechanism in adaptively modulating the influence of both the LSTM and RF models.

From the overall evaluation, the DW model emerged as the most effective among the models assessed, excelling in both predictive accuracy and computational efficiency. It matched or surpassed the RF and fusion models in terms of the MSE and MAE, while also achieving a high R value—demonstrating its capability to accurately model data trends. Notably, the DW model is distinguished by its exceptionally short training time, making it highly suitable for real-time prediction tasks where both speed and accuracy are essential.

Although the RF and fusion models also deliver strong predictive performance, their extended training times and lower computational efficiency limit their applicability in large-scale or time-sensitive scenarios. Although the LSTM and physical models offer moderate performance, their higher error rates in terms of the MSE and MAE make them inadequate for applications requiring high accuracy. Therefore, the DW model stands out as the optimal solution, delivering a balanced combination of accuracy and efficiency that is particularly advantageous in real-time prediction environments.

## 5. Conclusions

In this study, we introduced a time-series forecasting methodology that utilises multiple machine learning models to analyse data from the eLoran system. The models evaluated, including LSTM, RF, fusion, and DW, were comprehensively compared in terms of prediction accuracy, computational efficiency, and overall performance. The experimental results revealed that both the RF and DW models achieved high prediction accuracy while maintaining strong computational efficiency, positioning them as ideal candidates for applications requiring reliable real-time performance. The key findings and conclusions are summarised as follows.

### 5.1. Model Performance Comparison

The physical model, although excelling in computational speed, fell significantly short in prediction accuracy compared to more advanced machine learning models. Its simplicity makes it suitable for low-resource environments but inadequate for tasks demanding high precision. The LSTM model, with its deep learning architecture, is adept in capturing long-term dependencies in time-series data, yielding improved prediction accuracy.

However, its high computational requirements—evidenced by a training time of 73.91 s—limit its practical deployment in real-time or resource-constrained scenarios. The RF model outperformed the LSTM model in predictive accuracy, particularly for the ASF2 dataset, by effectively modelling nonlinear relationships in the data. It also surpassed the LSTM in predicting ASF1 values and demonstrated significantly better computational efficiency, with a training time of just 7.28 s.

The fusion model, which combines LSTM’s feature extraction with RF’s regression strength, achieved even greater prediction accuracy. Despite its extended training duration of 99.76 s—considerably longer than either RF or LSTM—the fusion model performed robustly, particularly when addressing complex time-series structures.

The DW model integrates the capabilities of LSTM and RF by dynamically adjusting their contribution weights based on the data characteristics. It achieved a remarkable training time of just 0.02 s, which was substantially lower than all other models, while maintaining high predictive accuracy. This balance of efficiency and performance makes the DW model especially well suited for real-time applications requiring both speed and precision.

### 5.2. Computational Efficiency Analysis

In terms of computational efficiency, the physical model exhibited the shortest training time at only 0.055 s, making it suitable for applications requiring rapid real-time responses. However, its limited prediction accuracy makes it impractical for applications where precision is critical. Conversely, the LSTM model, with a significantly longer training time of 73.91 s, excels in modelling long-term dependencies in time-series data. Despite its accuracy advantages, the model’s considerable computational demands restrict its applicability in real-time prediction scenarios.

The RF model offers a balanced approach, reducing the training time to 7.28 s while maintaining high predictive accuracy. This performance makes it an ideal choice for applications requiring prompt responses without a substantial compromise in accuracy. However, the fusion model integrates the training processes of both LSTM and RF, requires a significantly longer training time of 99.76 s, and involves greater computational complexity. Despite these challenges, it delivers enhanced prediction results through the integration of features from both models, making it valuable for complex data analysis tasks where accuracy is prioritised over speed.

The DW model presents an exceptionally efficient solution for time-series prediction, with a training time of merely 0.02 s—substantially shorter than the other models. By dynamically adjusting the contributions of LSTM and RF based on the data characteristics, the DW model not only achieves high predictive accuracy but also ensures a minimal computational overhead. This dual advantage makes it particularly well suited for real-time applications with stringent performance requirements.

### 5.3. Comparative Analysis of Forecasts

The visualisation results from the prediction comparison plot (Figure 9) and the dynamic weight adjustment plot (Figure 10) provide an intuitive understanding of each model’s predictive behaviour. The scatterplots reveal the alignment between the predicted and actual values across various models. Notably, the LSTM and RF models showed closer alignment with the actual values, especially in the ASF2 predictions, where the RF model demonstrated a superior fit.

The fusion and DW models exhibited further improvements, particularly in ASF2, where the prediction points were more tightly clustered along the diagonal, signifying high predictive fidelity. These results affirm the advanced capabilities of the fusion and DW models in capturing complex patterns within time-series data, with the DW model offering the added advantage of real-time responsiveness.

The dynamic weight change plot (Figure 10) depicts how the weights assigned to the LSTM and RF components fluctuated over time, reflecting the adaptive behaviour of the DW model. This plot reveals that the weights are dynamically adjusted at each timestep in response to the prevailing prediction errors, underscoring the model’s capacity to adaptively optimise its predictive contributions. By dynamically modulating the influence of LSTM and RF based on changing data characteristics, the DW model enhances the overall prediction accuracy and robustness, particularly in complex and variable environments.

### 5.4. Scenarios for the Application of Forecast Results

In this study, the effectiveness of various machine learning models was validated for time-series forecasting within the eLoran system, with the DW model emerging as the most effective. Through its dynamic weighting mechanism, the DW model improves the prediction accuracy while maintaining high computational efficiency, making it highly suitable for real-world implementation. In particular, systems that require the real-time processing of large-scale data with high precision—such as the eLoran system—can benefit substantially from the DW model’s capabilities.

In practical applications of the eLoran system, the forecasting methodologies developed in this study (including LSTM, RF, fusion, and DW models) enable the prediction of future ASF values using historical data and system features. These forecasts can support several critical operational functions.

① Adjustment of signal transmission power: If the predicted ASF values suggest potential signal attenuation or distortion, the system can proactively increase the transmission power to ensure consistent and reliable signal coverage.

② Selection of optimal signal path: In scenarios where multiple signal paths are available, ASF predictions can guide the system in selecting the most stable and optimal path, thereby improving the overall signal quality and reliability.

③ Dynamic adjustment of navigation accuracy thresholds: If forecasts indicate imminent and significant ASF fluctuations, the system can dynamically adjust its navigation accuracy thresholds to reduce the risk of positioning errors caused by sudden changes.

④ Failure prediction and early warning: Continuous ASF prediction facilitates the early detection of anomalies, enabling timely alerts and allowing technical personnel to implement preventative maintenance or corrective actions before a system failure occurs.

Although the results of this study demonstrate the strong predictive performance of the various models on the ASF1 and ASF2 datasets, we acknowledge that these datasets may not fully represent the diversity of eLoran operational environments. To address this limitation, as future work, we plan to expand our dataset collection to include additional scenarios with varying weather conditions (e.g., fog, rain), sea states (e.g., calm, rough seas), and geographical contexts (e.g., coastal areas, inland waterways, and port zones).

Moreover, we aim to evaluate the model’s robustness in more challenging settings, including high-noise, interference-prone, or weak-signal conditions that are common in real-world eLoran deployments. To further enhance the generalisation capabilities, we will explore domain adaptation and transfer learning techniques that allow models to adapt to unseen environmental features while maintaining predictive accuracy.

As additional future work, inspired by reinforcement learning principles, we plan to model the DW model’s weight assignment process as a sequential decision-making task. By treating the DW model as an agent interacting with a dynamic environment (i.e., varying signal quality), reinforcement learning techniques (e.g., Q-learning or policy gradient) may enable the model to learn adaptive, reward-driven weighting strategies that optimise the long-term prediction performance.

Although the primary focus of this study was models optimised for real-time implementation (e.g., LSTM, RF, DW), we acknowledge the potential advantages of advanced architectures such as transformer-based models and XGBoost. In future work, we plan to incorporate these models into our comparative analysis to assess their performance in terms of both prediction accuracy and computational efficiency. Transformer models are particularly powerful in modelling complex temporal dependencies, and XGBoost is known for its strong performance in structured data tasks. These additions will further enrich our understanding of model suitability under real-time constraints in eLoran systems.

In the present study, the ASF1 and ASF2 datasets were collected under relatively stable conditions. To acknowledge this limitation, we plan to conduct further experiments under more challenging environments. These include collecting data in interference-prone locations and introducing artificial noise to test the model robustness. We believe that this direction will enhance the comprehensiveness of the model evaluation.

## Figures and Tables

**Table 1:** Comparison of key model parameters.

Model	Fold	Train MAE	Val MAE	Train MSE	Val MSE	R (Validation)
LSTM	1	0.26	0.29	0.21	0.24	0.84
	2	0.25	0.28	0.2	0.23	0.86
	3	0.27	0.3	0.22	0.25	0.82
	4	0.26	0.29	0.21	0.24	0.85
	5	0.25	0.27	0.2	0.22	0.87
Fusion	1	0.23	0.26	0.18	0.22	0.86
	2	0.22	0.25	0.17	0.21	0.88
	3	0.23	0.26	0.18	0.22	0.85
	4	0.22	0.25	0.17	0.21	0.88
	5	0.22	0.24	0.17	0.2	0.89

**Table 2:** Performance and efficiency comparison of original vs. optimised fusion models.

Model Variant	LSTM Hidden Units	PCA Applied	Parallel Training	Train Time (s)	Val MAE	R (Val)
Original Fusion (LSTM + RF)	128	No	No	85	0.25	0.88
Optimised Fusion (LSTM + RF + PCA)	64	Yes	Yes	50	0.257	0.87

**Table 3:** Sensitivity analysis of the DW model with respect to smoothing factor α.

Smoothing Factor α	MAE (ASF1)	R (ASF1)	MAE (ASF2)	R (ASF2)	Weight Behaviour
0.05	0.087	0.970	0.092	0.965	Slow adaptation
0.10	0.082	0.974	0.088	0.969	Moderate, stable
0.20	0.079	0.976	0.084	0.970	Best performance
0.30	0.081	0.974	0.085	0.968	Fast response, some fluctuation
0.50	0.086	0.968	0.091	0.961	Unstable weights

**Table 4:** Performance comparison of ASF1 prediction accuracy.

Metric	Physical	LSTM	RF	Fusion	DW
MSE	168,265.57	2.21	0.48	0.51	0.37
MAE	30.20	0.73	0.47	0.50	0.46
R	0.13	0.44	0.68	0.69	0.73
Time	0.06	73.91	7.28	99.76	0.02
nMAE	291.47	7.05	4.54	4.83	4.44
nRMSE	1251.53	14.39	6.69	6.9	5.9

**Table 5:** Performance comparison of ASF2 prediction accuracy.

Metric	Physical	LSTM	RF	Fusion	DW
MSE	37.62	0.84	0.34	0.24	0.23
MAE	0.95	0.37	0.25	0.25	0.26
R	0.16	0.61	0.79	0.84	0.87
Time	0.06	73.91	7.28	99.76	0.02
nMAE	5.63	2.19	1.48	1.48	1.54
nRMSE	11.5	5.44	3.46	2.91	2.86

**Table 6:** Model deployment resource assessment.

Model	Computational Complexity	Memory Usage	Hardware Dependency	Power Consumption	Deployment Suitability
Physical (MLR)	Low	Very Low	Basic MCU or CPU	Very Low	Ideal for edge/embedded devices
Random Forest (RF)	Moderate	Low–Moderate	Embedded SoC or CPU	Low	Suitable for embedded/edge
LSTM Model	High	Moderate–High	High-performance CPU or GPU	High	Better for centralised/cloud systems
Fusion (LSTM + RF)	Very High	High	Multicore CPU and GPU recommended	High–Very High	Offline/cloud recommended
Dynamic Weighting (DW)	Moderate–High	Moderate	Adaptable to optimised CPU	Moderate	Edge-suitable with optimisation

## Data Availability

The data are not publicly available due to privacy.

## Abbreviations

The following abbreviations are used in this manuscript:

LSTM	Long short-term memory
RF	Random forest
DW	Dynamic weighting
GNSS	Global navigation satellite system
ASF	Additional secondary phase factor
BPNN	Backpropagation neural network
RNN	Recurrent neural network
MLR	Multiple linear regression
MSE	Mean squared error
MAE	Mean absolute error

**Notations**

$x_{t}$	Input feature at time step *t*
$h_{t}$	Hidden state at time step *t*
$h_{t-1}$	Hidden state from the previous time step
y	True value of the target variable
$\hat{y}$	Predicted value
*T*	Number of trees in the random forest
*N*	Number of samples in the dataset
*k*	Class index (in Gini calculation) or time step index (in optimisation)
$f_{t}(x)$	Prediction output from the *t*-th decision tree
$w_{\mathrm{lstm}}$ $,\;w_{rf}$	Weight coefficients for LSTM and RF predictions
⊙	Element-wise multiplication operator
$C_{t}$	Cell state at time step *t* in LSTM
$b_{f},b_{i},b_{C},b_{o}$	Bias terms for LSTM gates
$W_{f},W_{i},W_{C},W_{o}$	Weight matrices for LSTM gates
